# Pneumo-phono-articulatory coordination assessment in dysarthria cases: a cross-sectional study

**DOI:** 10.1590/1516-3180.2017.0320161217

**Published:** 2018-06-18

**Authors:** Rebeca de Oliveira Chappaz, Simone dos Santos Barreto, Karin Zazo Ortiz

**Affiliations:** I Speech-Language Pathologist, Department of Speech, Language and Hearing Sciences, Escola Paulista de Medicina, Universidade Federal de São Paulo (EPM-UNIFESP), São Paulo (SP), Brazil.; II MSc, PhD. Speech-Language Pathologist and Adjunct Professor III, Department of Specific Training in Speech, Language and Hearing Sciences, Instituto de Saúde de Nova Friburgo, Universidade Federal Fluminense (ISNF-UFF), Nova Friburgo (RJ), Brazil.; III MSc, PhD. Speech-Language Pathologist and Associate Professor IV, Department of Speech, Language and Hearing Sciences, Escola Paulista de Medicina, Universidade Federal de São Paulo (EPM-UNIFESP), São Paulo (SP), Brazil.

**Keywords:** Dysarthria, Speech disorders, Respiration, Speech production measurement, Neurology

## Abstract

**BACKGROUND:**

Pneumo-phono-articulatory coordination is often impaired in dysarthric patients. Because all speech is produced upon exhalation, adequate respiratory support and coordination are essential for communication. Nevertheless, studies investigating respiratory parameters for speech are scarce. The objectives of the present study were to analyze and compare the numbers of words and syllables (universal measurement) per exhalation among healthy and dysarthric speakers, in different speech tasks.

**DESIGN AND SETTING:**

A cross-sectional analytical study with a control group was conducted at the Department of Speech, Language and Hearing Sciences at UNIFESP.

**METHODS:**

The study sample consisted of 62 individuals: 31 dysarthric patients and 31 healthy individuals matched for sex, age and education level. All participants performed number counting and text reading tests in which the numbers of words and syllables per exhalation were recorded. All measurements obtained from the two groups were compared.

**RESULTS:**

Statistically significant differences between the dysarthric and healthy groups were found in the two tasks (counting of syllables and words per exhalation) (P < 0.001). In contrast, the performance of the dysarthric patients did not vary according to the task: reading and number counting in syllables/exhalation (P = 0.821) or words/exhalation (P = 0.785).

**CONCLUSIONS:**

The mean numbers of words and syllables per exhalation among dysarthric subjects did not vary according to the speech task used but they clearly showed differences between dysarthric patients and normal healthy subjects. The study also made it possible to obtain preliminary data on the average numbers of words and syllables per expiration produced by healthy individuals during their speech production.

## INTRODUCTION

Dysarthria refers to a group of speech disorders that arise from disruptions to the neuromotor control over muscle activities that are necessary for speech production. It occurs after damage to the central and/or peripheral nervous systems.^1^^,^^2^ Dysarthria can affect the performance of the pulmonary, laryngeal and pharyngeal structures as well as the oral and nasal cavities. Together, these provide the basis for phono-articulatory functions: respiration, phonation, resonance, articulation and prosody.^3^

With regard to respiration, a distinction in this function is made in the literature, between silent or vital respiration and respiration for speech. For phonatory activity, the higher the volume of air required, the greater the number of muscles involved. For speech, breathing takes place through recruitment of respiratory muscles, the skeletal musculature (controlled by nerve impulses) and the central nervous system, which allows release of the air current. This air current needs to generate sufficient air pressure to vibrate the vocal folds. Besides a large air volume, speech needs a slower respiratory rate and extended expiratory phase.^4^ Therefore, motor speech assessment of basal respiration in dysarthric patients often investigates vital capacity, respiration type and respiration rate (cycles per minute).^3^^,^^5^

Because all speech is produced upon exhalation, adequate respiratory support and coordination are essential for normal oral communication.^6^ Patients with damage to sensory or motor components of the respiratory system may have difficulty in maintaining adequate respiratory support for speech, as well as in coordinating exhalation with phonation and articulation.^7^ The breath group can serve as a functional unit for defining temporal features in continuous speech. These features of the breath group are determined by the physiological and linguistic demands of communication.^8^

There are speaking tasks that vary in these demands, such as number counting (from 1 to 20), reading and spontaneous speech,^3^^,^^9^ and these are commonly used to evaluate speech performance for research and clinical applications. These tests provide a quantitative and qualitative analysis by yielding objective measurements and the number of items produced per exhalation, and also allow investigation of individual speech. This is important because analysis on pneumo-phono-articulatory coordination also encompasses aspects such as use of residual air in utterances, sentence intelligibility and use of pauses at expected times and positions within utterances during a conversation.^6^^,^^10^ In fact, reduced mean length and variation of breath groups can cause inappropriate location of breath pauses that changes intonation and grammatical boundaries. Thus, these features reduce the intelligibility of speech and the communicative efficiency.^11^^,^^12^

Given the claims in the current literature that the locations and durations of breath groups are determined by physiological needs, linguistic accommodations and cognitive demands,^10^^,^^11^ it is worth mentioning that these features can differ across speaking tasks and language spoken. In Brazil, no data on the number of words per exhalation is available. However, a normative value of 25 phrase elements per breath among Portuguese speakers has been proposed in a book, without specifying the speech task used.^13^ Regarding individuals with speech disorders, a Brazilian study on 60 dysarthric patients found a mean of 7.7 words per breath for number counting and 6.8 words per breath in spontaneous speech, but there were no data for healthy speakers regarding these tasks.^3^ Although words per breath is a valuable measurement, especially considering the linguistic approach, it is not meaningful for international comparisons. In this regard, measurements based on syllable units have been recommended.^5^ Considering the importance of respiration in relation to speech and its implications for diagnosis and rehabilitation of dysarthric patients, studies are necessary in this field, taking into account the different tasks and the Portuguese language.

The aim of the present study was to analyze the number of words and syllables per exhalation among speakers with and without speech disorders, in two tasks assessing pneumo-phono-articulatory coordination (number counting and text reading), and to compare the performance of dysarthric speakers with the performance of non-dysarthric individuals in these two tests by analyzing the numbers of words and syllables per exhalation in these groups.

## METHODS

The present cross-sectional study was carried out in the Department of Speech, Language and Hearing Sciences at the Federal University of São Paulo (Universidade Federal de São Paulo). The study had previously been approved by this institution’s research ethics committee (permit number 0069/11).

The sample comprised a patient group of 31 individuals with dysarthria who had previously been assessed at the Neuropsycholinguistics Laboratory and a control group of 31 healthy individuals who were matched for sex, age and education level. Body type was not controlled for in this study, since there is no consensus regarding whether body type influences speech breathing.^14^ Moreover, a more recent study with a larger sample has suggested that there is no difference between speech tasks (counting and reading) and body type (endomorphy, mesomorphy or ectomorphy).^15^

The dysarthric group consisted only of native speakers of Brazilian Portuguese with a single diagnosis of dysarthria acquired in adulthood and a medical diagnosis of neurological disorder. All the patients performed the tests to assess the number of words per exhalation in the number-counting and text-reading tasks of the dysarthria protocol.^5^^,^^16^^,^^17^ Individuals with other speech, language and/or cognitive disorders that were investigated during the overall neuropsychological assessment were excluded from the study. The data relating to the dysarthric group were collected from patients who had previously been evaluated at the outpatient clinic for speech and language neurological disorders.

The control group included only Brazilian-Portuguese native speakers who were companions or family members of the patients assessed at the Neuropsycholinguistics Laboratory. The general exclusion criteria were as follows: history of alcoholism or drug use, history of communication disorders, current or previous neurological and/or psychiatric diseases, use of psychotropic medications and absence of visual or auditory impairments that might affect the outcome from the tasks. Data relating to the control group were prospectively collected in accordance with the matching proposed in the study design. All of these data were obtained by the same examiner under the same professional supervision.

Upon application of the tests, the subjects were first instructed, after one inspiration, to start counting from 1 to 20 aloud at their natural speech rate and to pause for breath as many times as necessary to finish counting. Then a text that had been written in a standard format and typed using the font Arial 14 was given to each subject. They were asked firstly to read it through to become familiar with the story and then to read it again, out aloud at their usual speed of reading. The text used for this evaluation comprised 129 words, which is the average number that has been suggested in many international protocols^17^^,^^18^ (Appendix 1 ^16^).

The number of inhalations made during the two tasks was counted, from the first inhalation prior to the counting and reading tasks, to the last one made that was made just before the end of the tasks. The number of words and the number of syllables produced were then divided by the total number of inhalations.

Wilcoxon’s nonparametric test (5% significance level) was applied to compare the performance of the dysarthric group regarding the numbers of syllables and words in the two tasks. Matching and comparison of the two groups (healthy and dysarthric) in each speech task, regarding the numbers of syllables and words, was performed using the Mann-Whitney test at the 5% significance level.

## RESULTS

### Sample characteristics

The two groups were matched for sex, age and education level. The sample consisted predominantly of men (68%), such that each group comprised 21 men and 10 women. The variables of age and education (numbers of years of schooling with approval to pass to the next level), along with means and standard deviations, are shown in Table 1. Because the samples were matched, there was no statistically significant difference between the groups regarding age, sex and schooling years.

**Table 1:** Sample characteristics regarding the variables of sex, age and education level

	Classification		P-value
	Dysarthric	Healthy	
Sex (n; %)			
Female	10 (32.3%)	10 (32.3%)	1.000*
Male	21 (67.7%)	21(67.7%)	1.000*
Age (years)			
Mean	50.9	50.3	0.877**
SD	17.7	17.5	
Education level (years)			
Mean	7.4	7.4	0.836**
SD	4.1	4.5	
N	31	31	

SD = standard deviation. *Chi-square test; **Mann-Whitney test; P ≤ 0.05.

With regard to the etiology of the dysarthric patients, 16 (51.6%) had the non-progressive type of dysarthria: fourteen had suffered a stroke and two had had a traumatic brain injury. Fifteen (48.3%) presented progressive etiology: three had amyotrophic lateral sclerosis, one had Huntington’s disease, five had Parkinson’s disease, 5 had ataxia (different types) and one had dystonia. Regarding the frequency distribution of dysarthria types, the most prevalent was mixed (25.8%) followed by hypokinetic (22.6%), upper motor neuron (16.1%), flaccid (12.9%), spastic (12.9%), hyperkinetic (6.5%) and ataxic (3.2%).

The means and standard deviations for the numbers of words per exhalation (WPE) and syllables per exhalation (SPE) in the number counting and text reading tasks, together with the comparison between the two groups (values from the Mann-Whitney test), are shown in Table 2. Dysarthric patients performed worse than healthy controls.

**Table 2:** Comparison between numbers of words and syllables per exhalation produced by dysarthric patients and healthy individuals in number-counting and text-reading tasks

	Classification		Mann-Whitney test (P)	Result
	Dysarthric	Healthy		
Number counting (WPE)				
Mean	5.1	11.3	< 0.001*	Dysarthric < Healthy
SD	4.4	7.4		
N	31	31		
Number counting (SPE)				
Mean	10.7	23.2	0.001*	Dysarthric < Healthy
SD	9.3	15.3		
N	31	31		
Text reading (WPE)				
Mean	5.7	8.5	0.001*	Dysarthric < Healthy
SD	4.4	4		
N	31	31		
Text reading (SPE)				
Mean	11.9	17.8	0.001*	Dysarthric < Healthy
SD	9.1	8.4		
N	31	31		

WPB = words per breath; SD = standard deviation; SPE = syllables per exhalation. Mann-Whitney test (P ≤ 0.05).

In order to ascertain whether the two tasks were equally useful for identifying impairments in pneumo-phono-articulatory coordination, the performance of individuals with dysarthria was compared between the two tasks and between the two measurements (syllables and words). There was no statistically significant difference in the number of words or number of syllables per exhalation produced by the dysarthric group in the two tasks (Table 3).

**Table 3:** Comparison of number of words per exhalation (WPE) and of syllables per exhalation (SPE) produced on tasks in dysarthric group

	Counting	Reading	Wilcoxon’s test (P)	Result
WPE				
Mean	5.1	5.7	0.785	counting = reading
Median	3.0	4.7		
Minimum	1.0	1.0		
Maximum	20.0	21.0		
Standard deviation	4.4	4.4		
n	31	31		
SPE				
Mean	10.7	11.9	0.821	counting = reading
Median	6.3	9.8		
Minimum	2.1	2.1		
Maximum	42.0	43.6		
Standard deviation	9.3	9.1		
n	31	31		

## DISCUSSION

The main finding from this study was that the speech breathing tests (number-counting and reading), using words or syllables as the parameter, were sensitive for identifying alterations of respiration, which are one of the motor components of dysarthria. This study showed clear differences between dysarthric patients and normal healthy subjects. In addition, we were able to obtain data that can be used as clinical reference values for speech breathing assessment. Another relevant finding was that the performance of the dysarthric group did not differ between the two tasks. These and other results are discussed further below.

Regarding sample characterization, the most frequently found etiology among the patients was cerebrovascular disease, and there were more males than females, thus corroborating the findings from previous studies.^19^^,^^20^ Although there were more males in our sample, previous studies have observed no difference in speech breathing between the sexes.^21^ Regarding schooling, the mean duration was found to be 7.4 years, equivalent to incomplete elementary school. Studies have shown that in Brazil, the users of the public healthcare system still predominantly have low literacy levels.^22^^,^^23^

Age also constitutes an important factor in brain lesions. The patients’ mean age was 50.9 years (Table 1). Younger individuals are expected to be less vulnerable to risk factors that can cause neurological lesions, whereas older adults may present greater numbers of associated risk factors,^22^ although there is no consensus in this regard in the literature.^24^^,^^25^ Moreover, the age at the onset of degenerative conditions is highly variable.

The most prevalent form of dysarthria, occurring in 25.8% of the patients, was the mixed type. In this, individuals exhibit the combined characteristics of different forms of dysarthria and have lesions involving multiple areas of the central and/or peripheral nervous system, as occurs in degenerative diseases. It is important to point out that the most common etiology among our patients was non-progressive and the most frequent cause was stroke.

The number of words per exhalation in the two tasks among the dysarthric individuals was lower than values previously reported in the Brazilian literature, which were 7.7 words per exhalation in the counting task and 6.8 in the text-reading task (Table 2). As mentioned previously, no data for these tests are available.^3^ The findings from this previous study cannot easily be compared with those of the present study, because there are different types and degrees of motor speech disorders that compromise respiration, phonation, articulation, resonance and prosody in many ways in dysarthric patients. For sensory-motor evaluation of speech, it is important to understand how each deficit in any motor component can impact speech production, and intra and interarticulatory factors need to be extensively examined.

The values from individuals without speech disorders, shown in Table 2, are helpful for establishing the magnitude of the deficit (in comparison with patients) and for following up the rehabilitation process.^26^ Rehabilitation of speech encompasses all motor bases, including respiration, which underpins the other ones. The primary function of breathing is gas exchange (quiet respiration), but breathing also generates airflow and pressure to produce the voice and speech.

Breathing for the speech function is a refinement of vital respiration, in which individuals use around 20% of the total volume of the lungs, compared with around 10-15% for quiet respiration. There is also a difference in respiratory rate, such that it is slower in speech, averaging eight cycles per minute, compared with 16-18 cycles in quiet respiration. Another important difference is exhalation during speech, which can be up to forty seconds long, while inhalation accounts for only 10% of the total respiratory cycle, whereas the ratio of the breathing phases for quiet respiration is 1:1.^4^ There is a difference in breathing for these two functions.

As shown in Table 2, there was a difference in coordination between respiration and phonation, between the dysarthric and control groups. For speech production, greater intensity of neural motor refinement is required, such that coordination of breathing is fundamental for voice and speech production. Moreover, motor control for air inspiration and volume, depth of inspiration and control of expiration needs to be taken into consideration.^6^ Determining how speech tasks affect breath group organization is important, because these tasks are often an integral part of the clinical assessment battery that is used to evaluate dysarthria.^8^^,^^16^ In addition, understanding of breath group patterning is important for improvement of naturalness of speech. Furthermore, proper intonational variations within the breath group provide listeners with cues about linguistic and grammatical structures.^8^^,^^11^^,^^12^

No statistically significant difference in the numbers of words or syllables per exhalation was found among the dysarthric patients (Table 3). This was probably because the tasks and the measurements are probably equally sensitive for making the diagnosis, given that pneumo-phono-articulatory coordination is impaired in dysarthric patients.

There is a relationship between lung volume and duration of utterance, in which the magnitude of the lung volume is influenced by the length of the utterance to be produced. In reading, the grammatical structure of the utterance is a factor that influences pauses.^27^ Thus, during text reading, syntactically determined inspirations occur, i.e. inspirations dictated by the text structure. Consequently, there is a relationship between the relative amount of air inspired and the location of the syntactic pauses, thus leading to some expected inspiration during reading.

Utterances are influenced not only by type and syntactic structure, but also by voice quality and intensity and the oral projection required. These factors can lead to different results from the tasks. However, the discourse of individuals with dysarthria contains more pauses between and within phrases, and may occur within words in more severe cases. Such pauses are incongruent and indicate pneumo-phono-articulatory incoordination among these speakers.^7^

Although the two tasks, reading and counting, differ and can assess different speech abilities, they are equally sensitive for measuring the number of words per exhalation, with regard to assessing basal breathing and pneumo-phono-articulatory coordination. This information may be especially useful in situations in which reading cannot be applied, such as in cases among patients with visual deficits or who are illiterate or have low literacy levels. Thus, a task that makes use of automatism, and which can reliably assess pneumo-phono-articulatory coordination and is independent of educational level, is a useful alternative for speech breathing assessment.

The limitations of this study were, firstly, that it was a cross-sectional study. Thus, although the tasks could identify differences between dysarthric and normal subjects, the data obtained from normal subjects should not be understood as normative but only as preliminary. Secondly, patients with different types of dysarthria were evaluated. Further studies should be conducted in order to more precisely investigate the impact of respiration on speech production in situations of different types and degrees of dysarthria. In addition, more studies should investigate speech breathing among normal healthy subjects, taking different linguistic tasks into consideration.

## CONCLUSION

The mean numbers of words and syllables per exhalation among dysarthric individuals were the same in the two tasks used (automatism and text reading), but the values for the patients differed significantly from those of the healthy individuals. Both of these tasks are useful for speech breathing assessments among dysarthric patients.

## Appendix 1

Text used in reading task^16^

Um homem velho, que vivia sozinho há muito tempo, não suportava crianças. Ele morava numa casa grande e mantinha uma vara de bambu ao alcance de sua mão, com a qual ameaçava as crianças de um prédio BHN vizinho. Um dia, quando ele estava destruindo um ninho de pardais, ficou preso sobre o telhado alto de três metros e cinquenta. Isso porque, querendo descer muito rápido, deixou cair a escada que tinha colocado mal equilibrada contra a parede do sobrado. Como o homem começou logo a gritar, um garoto corajoso, que brincava calmamente na rua, levantou a cabeça, compreendeu a situação e recolocou a escada caída no chão ao lado de uma roseira. Depois dessa vergonhosa aventura, ele ofereceu ao menino um lanche acompanhado de suco de maçã.
